# Automated Measurement of Patient-Specific Tibial Slopes from MRI

**DOI:** 10.3390/bioengineering4030069

**Published:** 2017-08-08

**Authors:** Amirhesam Amerinatanzi, Rodney K. Summers, Kaveh Ahmadi, Vijay K. Goel, Timothy E. Hewett, Edward Nyman

**Affiliations:** 1 Engineering Center for Orthopaedic Research Excellence (ECORE), Departments of Bioengineering and Orthopaedic Surgery, Colleges of Engineering and Medicine, The University of Toledo, Toledo, OH 43606, USA; Amirhesam.Amerinatanzi@rockets.utoledo.edu (A.A.); Rodney.Summers@rockets.utoledo.edu (R.K.S.); Kaveh.Ahmadi@rockets.utoledo.edu (K.A.); Vijay.Goel@utoledo.edu (V.K.G.); 2 Mayo Clinic Biomechanics Laboratories and Sports Medicine Center, Departments of Orthopedics, Physical Medicine and Rehabilitation and Physiology & Biomedical Engineering, Mayo Clinic, Rochester, MN 55905, USA; Hewett.Timothy@mayo.edu; 3 Departments of Health and Human Performance & Physical Therapy, College of Health Professions, The University of Findlay, Findlay, OH 45840, USA

**Keywords:** knee, tibial slope, concavity, MRI

## Abstract

Background: Multi-planar proximal tibial slopes may be associated with increased likelihood of osteoarthritis and anterior cruciate ligament injury, due in part to their role in checking the anterior-posterior stability of the knee. Established methods suffer repeatability limitations and lack computational efficiency for intuitive clinical adoption. The aims of this study were to develop a novel automated approach and to compare the repeatability and computational efficiency of the approach against previously established methods. Methods: Tibial slope geometries were obtained via MRI and measured using an automated Matlab-based approach. Data were compared for repeatability and evaluated for computational efficiency. Results: Mean lateral tibial slope (LTS) for females (7.2°) was greater than for males (1.66°). Mean LTS in the lateral concavity zone was greater for females (7.8° for females, 4.2° for males). Mean medial tibial slope (MTS) for females was greater (9.3° vs. 4.6°). Along the medial concavity zone, female subjects demonstrated greater MTS. Conclusion: The automated method was more repeatable and computationally efficient than previously identified methods and may aid in the clinical assessment of knee injury risk, inform surgical planning, and implant design efforts.

## 1. Introduction

Anterior-posterior (AP) stability and translation of the knee joint is checked, in part, by the posterior tibial slope (PTS) [1,2,3,4,5] which has been identified as a risk factor for non-contact ACL injuries in otherwise healthy individuals [1,5,6,7,8,9]. This increase in risk is most likely attributable to increased mechanical tension on the cruciate ligaments through a direct, or indirect, effect on tibiofemoral joint kinematics. Hashemi et al. [10] suggested differences in the role the medial and lateral PTS play in tibiofemoral kinematics, with the lateral slope having a greater impact risk of ACL injury when compared to the medial slope, especially in females [7].

Various methods for measurement of the tibial slope, in either the sagittal or coronal planes, have been previously established and well documented in the literature [10,11,12,13,14,15,16,17]. It has also been acknowledged that 2D radiographic images are not suitable for quantifying PTS since differences in medial and lateral tibial morphology are ignored [10,12]. Between the work done by Hashemi et al. [10,12] and Giffin et al. [5], a method for determining tibial slope has been established based on the use of a reference axis and planar (sagittal and coronal) measurements [5,12].

Although these efforts have contributed largely to the advancement of the understanding of knee joint arthrokinematics, these methods are not without error. Even when trained observers follow established protocols, unavoidable variability is introduced, engendering errors in the final reported tibial slopes [18,19]. This error can be credited to the subjective nature of the inherent procedural methodology concomitant with error affiliated with 2D imaging [12]. As subjective visual assessment has traditionally been used to pick out the center of the diaphysis, the collective errors associated with the 2D approaches can be attributed to the following: (1) error in choosing the appropriate image slice; (2) error in selecting precise anatomical landmarks to measure tibial geometry; and (3) assumptions of constant tibial geometry (i.e., plateau slope and diaphyseal axis orientation). Accurate and reproducible measurement of the proximal tibial anatomical axis is therefore confounded via a 2D-based approach [9]. Inconsistencies in slice selection and slice thickness can compound error when defining the tibial slope. Another weakness of the traditional 2D approaches is the inability to accurately identify points of interest (i.e., anterior and posterior borders of the plateau).

Historically the PTS has been defined as the angle between the tangent of the medial or lateral tibial condyles and a line perpendicular to the mechanical axis, or a longitudinal axis, of the shank segment [3,10,12,19,20,21,22]. The method commonly used to define this axis inherently introduces error and may yield inconsistent results [23]. A study by Sheehy et al. established that the placement of the reference axis can vary between observers based partly on the amount of the distal tibia observable in the magnetic resonance images (MRI) [23]. Choosing an appropriate sagittal or coronal reference plane from which to measure the tibial slope is of concern. The methods established by Hashemi et al. and Giffin et al. rely on bisecting two lines made in the distal portion of the tibia to define this reference axis [5,10,12]. The tibial slopes are defined as the angle between the normal to the reference plane and a line connecting the AP borders for medial and lateral posterior tibial slopes (MTS and LTS, respectively) or the medial-lateral (ML) borders for coronal tibial slope (CTS). A study by Faschingbauer et al. demonstrated that tibial slope can vary up to 7.5° depending on the length of the diaphysis observable in the image [19]. Such scan length dependence can lead to inaccurate representation of the tibial axis and produce potentially misleading slope measurements.

In this study, an automated approach to a methodology previously published by the authors was evaluated [24,25]. The main objectives of this study were to evaluate computational efficiency, reliability, and repeatability of the automated method. Efforts were directed at enhancing the objectivity of tibial slope measurement while eliminating dependence on image scan length. The proposed automated method was evaluated for both sagittal and frontal plane MRI scans. In addition to our effort in developing this new method, we hypothesized that: (1) LTS and MTS would vary significantly across the tibial plateau; (2) LTS and MTS would vary between subjects; and (3) LTS and MTS would vary within subjects (between left and right sides). 

## 2. Materials and Methods

Following Intitutional Review Board (IRB) approval in accordance with the World Medical Association Declaration of Helsinki, 3.0 Tesla MRIs of the proximal tibia, 1.6 mm slice thickness with 0.8 mm gap, were obtained from nine subjects and uploaded to Mimics 15.0 (Materialise, Leuven, Belgium). All data was de-identified in compliance with Health Insurance Portability and Accountability Act (HIPAA) to ensure subject confidentiality. The subjects were positioned supine, unloaded, and neutrally aligned during collection of frontal, sagittal, and axial images. A custom code was developed in Matlab (Mathworks, Natick, MA, USA) to locate MRI slices in Mimics, perform curvature analysis, and calculate slope measurements from each slice. Additionally, the code was provisioned to define a “concavity zone” of potential arthrokinematic interest. The concavity zone was defined as the region of the tibial plateau bound by the transverse plane and the superior nodes (edges). More details on the concavity zone are presented in Section 2.3. 

### 2.1. Medial and Lateral Tibial Slope Measurement Methodology

**Figure 1:**
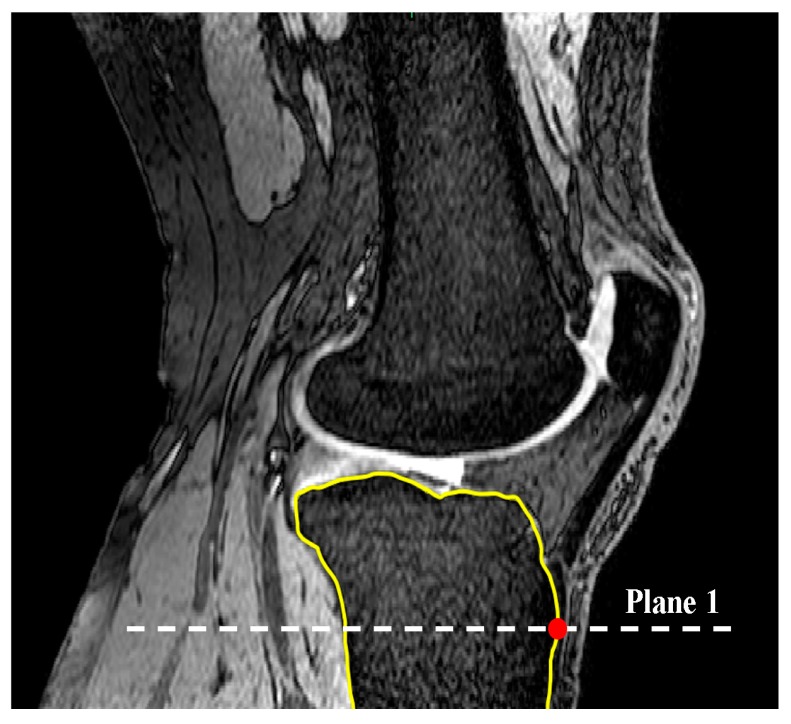
Identification of the most anterior point on the tibia in the sagittal plane to define plane 1 (Sagittal view).

**Figure 2:**
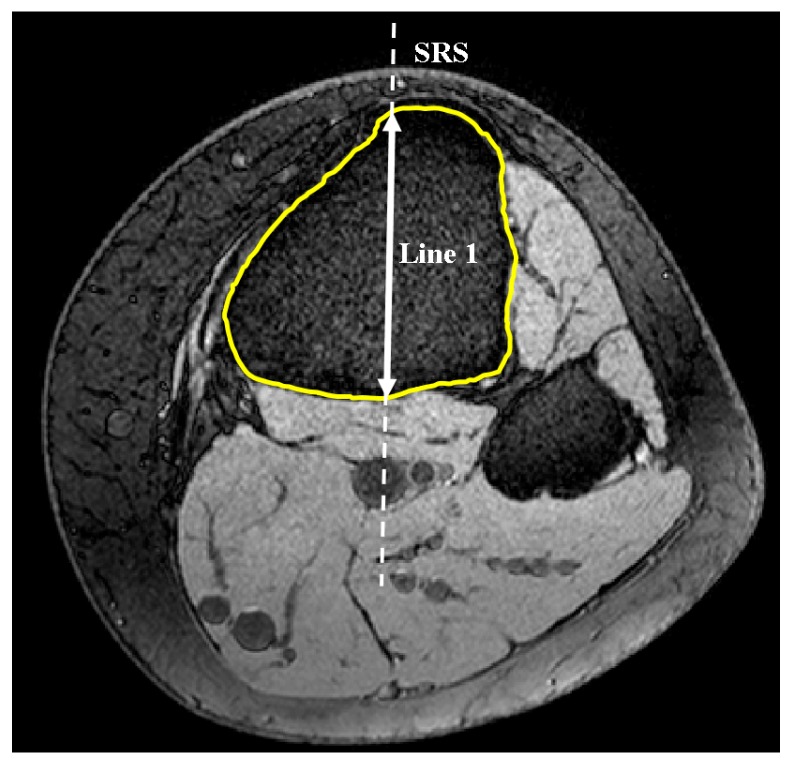
Axial view. Determination of the longest AP line constrained to tibial border in plane 1 and therein defining SRS (axial view).

The code automatically identified the most anterior point on the tibial tuberosity and generated a transverse plane (plane 1) that passed through this point (Figure 1). Successive lines produced from the intersection of plane 1 and MRI sagittal slices were then set in the anterior-posterior (AP) direction in plane 1 in order to define the longest cross-section of the tibia in this direction (Figure 2). This sagittal plane slice, within which line 1 was located, was defined as the sagittal reference slice (SRS).

**Figure 3:**
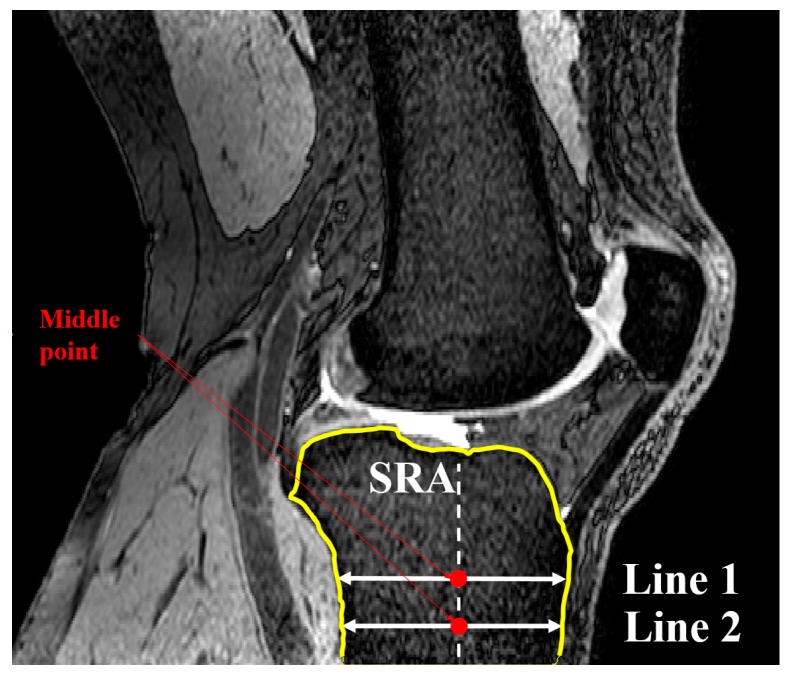
Drawing line 2 in the SRS to define SRA (sagittal view).

**Figure 4:**
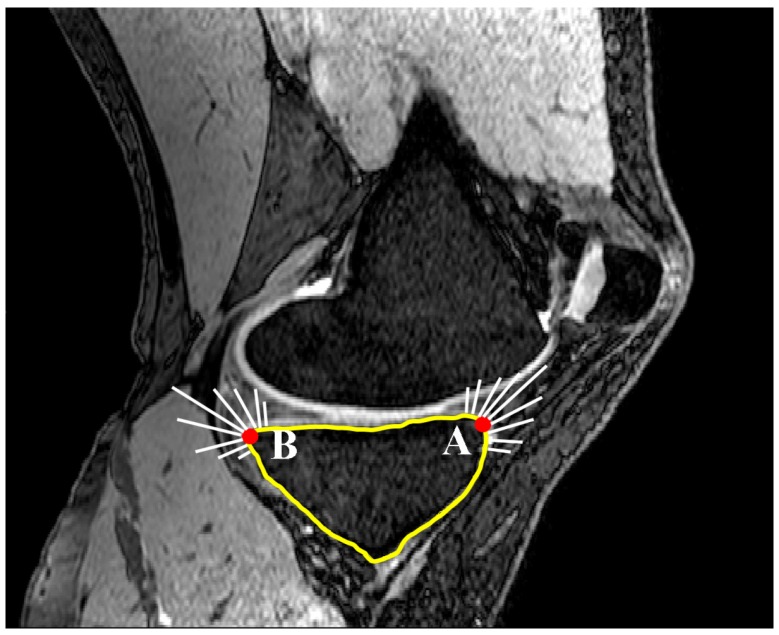
Locating nodes A and B based on Gaussian curvature analysis for each MRI slice (sagittal view).

**Figure 5:**
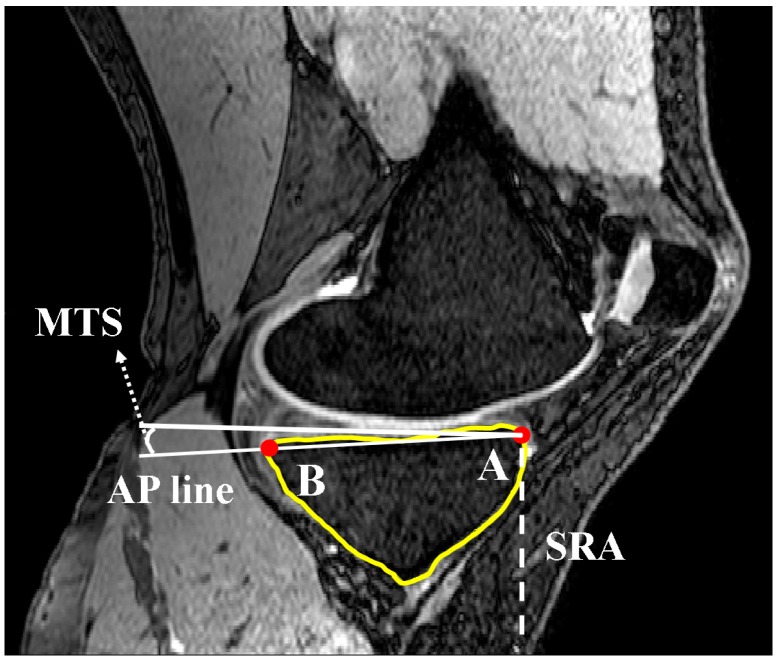
Measurement of posterior tibial slope as the angle between AP line and the SRA (sagittal view).

A second line (line 2) was set parallel and 10 mm distally to the initially defined line in the SRS. Lines 1 and 2 were limited to the anterior and posterior borders of the tibia through contours generated within Mimics. The contours were defined by generating two automatic threshold masks and using a Boolean subtraction to leave behind the contour on the cortex of the bone. A sagittal reference axis (SRA) was defined by connecting the midpoints of lines 1 and 2 (Figure 3). The anterior and posterior points of the tibia plateau in each slice were then identified using Gaussian curvature analysis (Figure 4). The nodes (A and B) with the greatest curvature at the anterior and posterior borders of the tibial plateau in each slice of interest were then defined and a line (AP line) was introduced between the nodes such that a line perpendicular to the SRA and crossing node A could be drawn. Tibial slope was defined as the angle between the AP line and the aforementioned perpendicular line (Figure 5). If point B fell below the line perpendicular to the SRA, the slope was reported as positive.

Finally, the distance of each slice from the SRS was normalized to the maximum distance of the lateral or medial aspects of the plateau with respect to the SRS such that intra- and inter-subject data could be appropriately compared.

### 2.2. Coronal Tibial Slope Measurement Method

**Figure 6:**
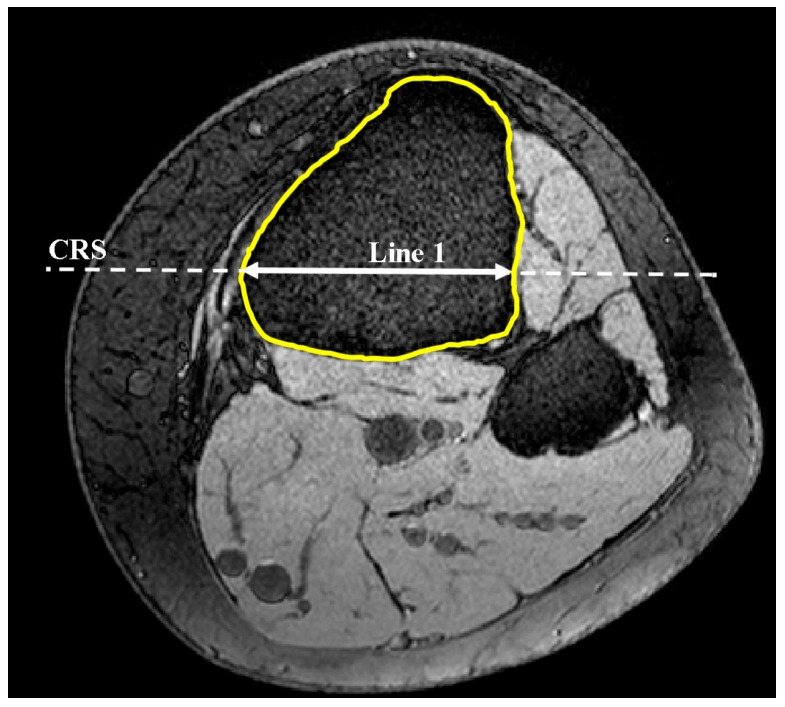
Identification of the longest medial-lateral line limited to tibial border in plane 1 and therein defining CRS (axial view).

**Figure 7:**
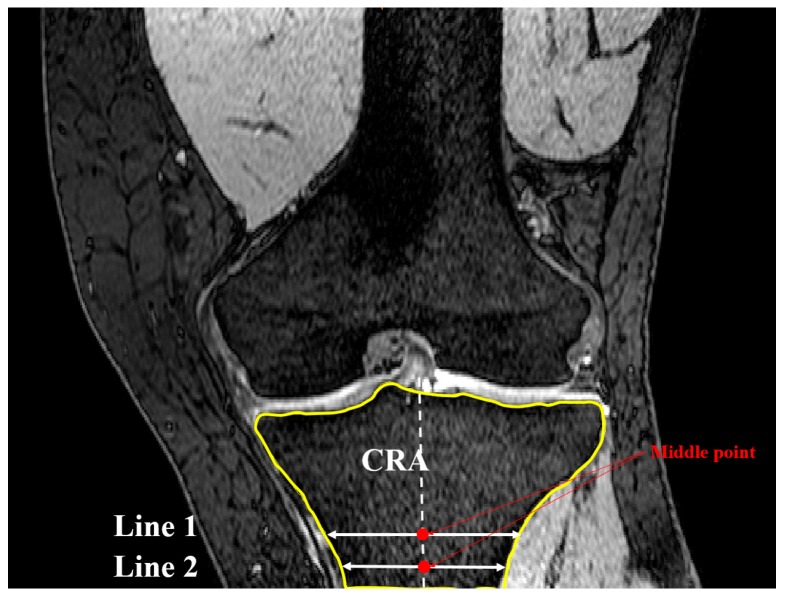
Definition of the second line parallel and 10 mm distal to line 1 in CRS and therein defining CRA (coronal view).

The coronal plane tibial slope method was nearly identical to sagittal slope measurements, with the exception being the definition of the reference planes and axis. Here, a transverse plane (plane 1) was passed through the most anterior point of the tibial tuberosity as shown previously in Figure 1. Lines, produced from the intersection of plane 1 and MRI frontal slices, were drawn to identify the greatest medial-lateral cross-section within the tibial borders of plane 1 (line 1). The frontal plane (Figure 6) that included line 1 was deemed the coronal reference slice (CRS). A second line was introduced in the CRS 10 mm distally and parallel to line 1 constrained to the tibial cross-section. The midpoints of these lines were connected to define the coronal reference axis (CRA) as depicted in Figure 7.

**Figure 8:**
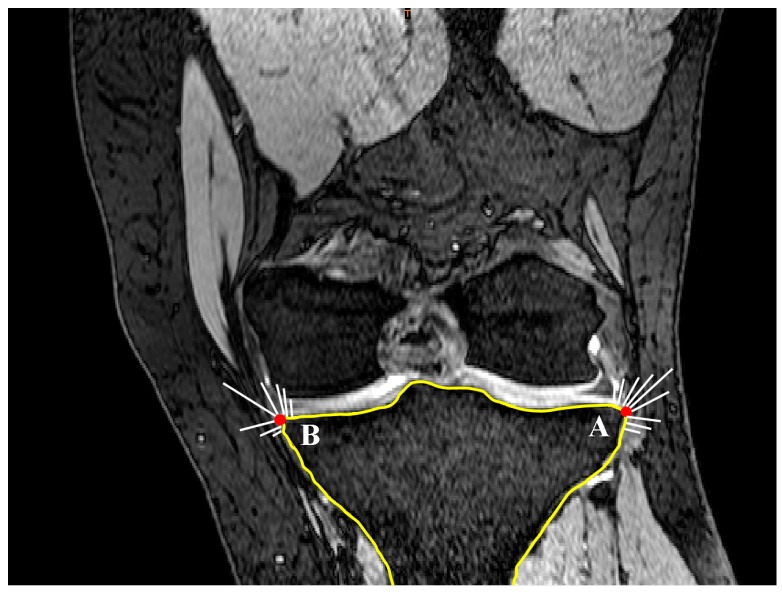
Location of nodes A and B based on Gaussian curvature analysis (coronal view).

**Figure 9:**
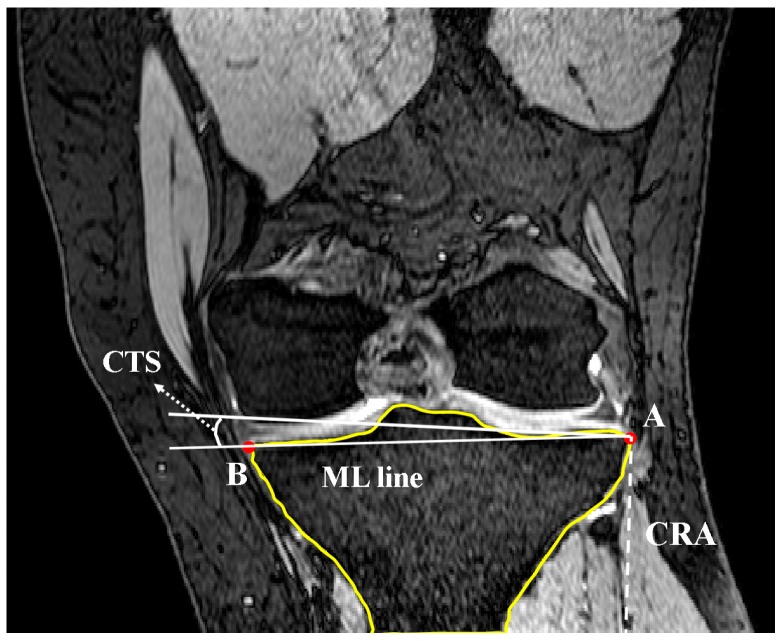
Calculation of coronal tibial slope in interested MRI slice as the angle between ML line and the perpendicular line of CRA (coronal view).

The Gaussian curvature analysis was employed again to identify the points on the medial and lateral aspects (nodes A and B in Figure 8) with the greatest curvature. The coronal tibial slope (CTS) was defined as the angle between the line connecting nodes A and B (ML line) and the line perpendicular to CRA and crossing node A (Figure 9). If point B was below the perpendicular line, slope was reported as positive. This measurement was repeated for successive slices across the tibial plateau.

The distance of each frontal plane MRI slice from the CRS was normalized with respect to the distance from the CRS to either the most anterior or most posterior aspect of the proximal tibia such that intra- and inter-subject data could be appropriately compared.

### 2.3. Concavity Zone

**Figure 10:**
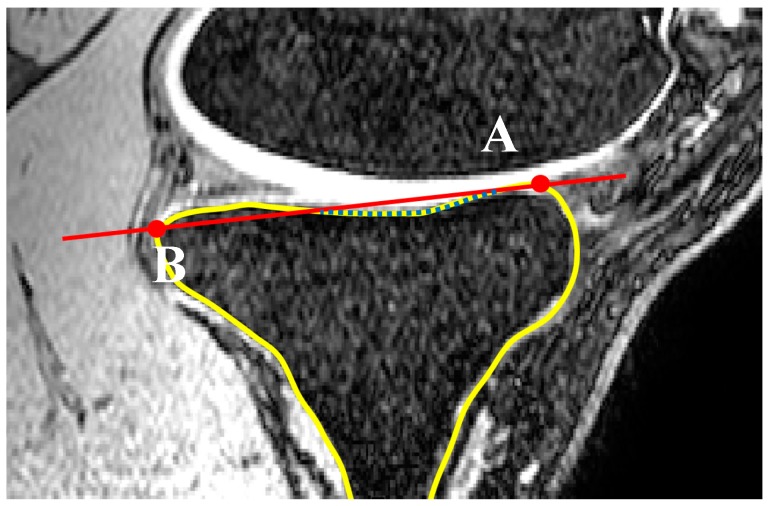
Definition of nodes placed on the concavity in a sagittal slide (dotted line). Nodes A and B are defined based on curvature analysis (sagittal view).

**Figure 11:**
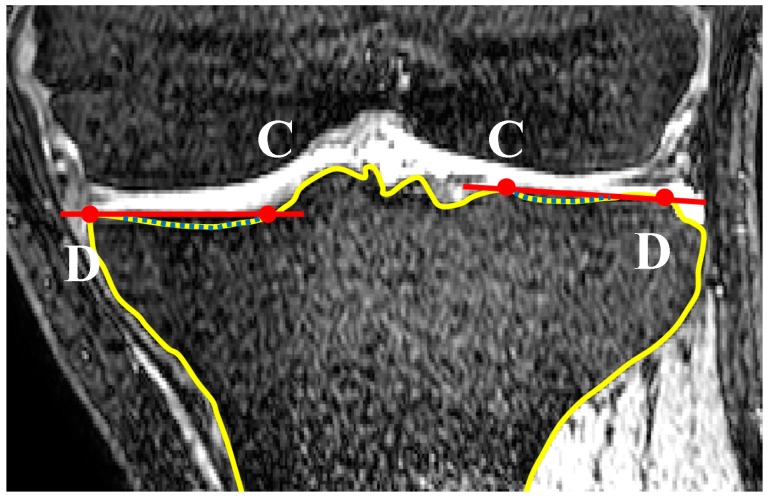
Definition of nodes placed on the concavity in a coronal slide (dotted line). Nodes C and D on both lateral and medial aspects are defined based on curvature analysis (coronal view).

**Figure 12:**
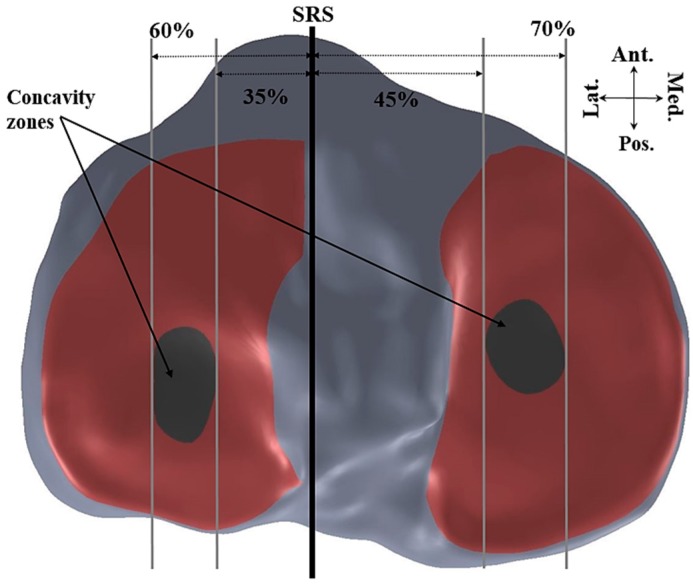
Definition of the concavity zones based on lateral and medial distance to SRS (axial view).

Curvature analysis was used to define the edges of the medial and lateral tibial plateaus. Nodes A and B in the sagittal plane (Figure 10) and nodes C and D in the coronal plane (Figure 11) were defined in each of the sagittal and coronal planes. Then the nodes on each plateau were compared with the connecting lines (AB for sagittal slides and CD for the coronal slides) and any nodes inferior to these were defined as nodes of the concavity zone (Figure 12).

## 3. Results

The thresholds of the concavity of the lateral and medial tibial plateaus were defined based on Gaussian curvature analysis. The lateral concavity zone was limited between 35–60% of the maximum lateral distance from the SRS and the medial concavity zone was limited to 45–70% of the maximum medial distance from the SRS. Mean tibial slopes were then calculated along each plateau and each concavity zone.

**Figure 13:**
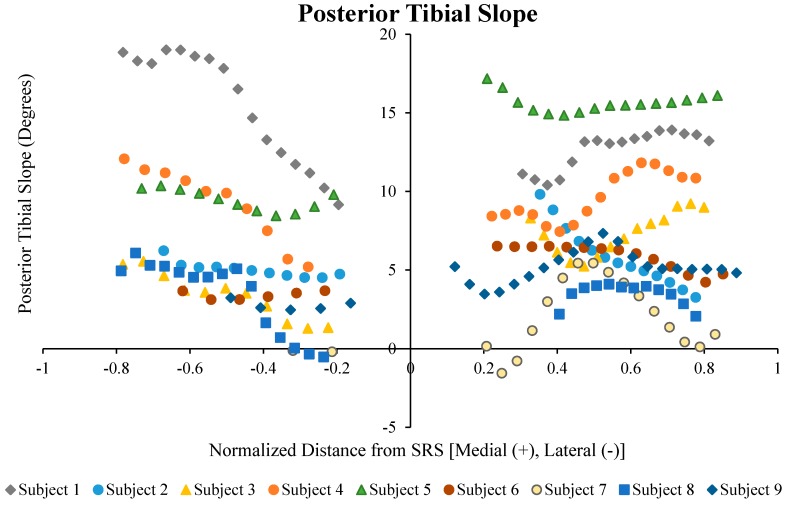
Tibial slopes across the lateral and medial plateaus normalized to the maximum distance to the medial or lateral aspect of the plateau from the sagittal reference slice for each subject.

A summary of the initial calculation of each subject is presented in Figure 13 and Table 1. The correlation coefficients between the coronal tibial slope and medial tibial slope, and the coronal tibial slope and lateral tibial slope were not significant for between sex and across the sample population (*p* > 0.1). The results indicate that the mean LTS for females (7.2°) was greater than that for males (1.66°). Moreover, the mean LTS in the lateral concavity zone for the female subjects was greater than for male subjects (7.8° for females vs. 4.2° for males). The mean MTS in the female subjects was greater than that of male subjects (9.3° vs. 4.6°, respectively). Furthermore, along the medial concavity zone, female subjects had greater MTS compared to male subjects.

**Figure 14:**
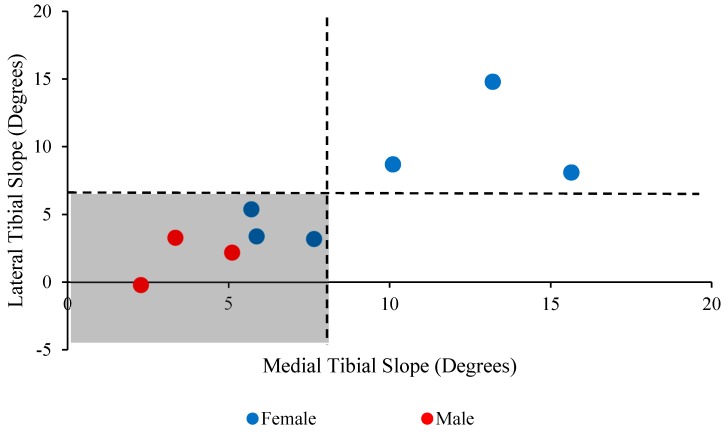
Medial tibial slope vs. lateral tibial slope values for male and female subjects. Data shows a greater concentration (66.7%) of the subjects in the highlighted zone.

Figure 14 shows a greater distribution (66.7%) of the lateral and medial tibial slopes between the populations in the highlighted zone (i.e., the medial and lateral tibial slopes are less than 8° and 5.5° in the lower left quarter, respectively).

## 4. Discussion

Investigation of the sagittal and frontal plane tibial slopes was done in this study. The main hypotheses were that the medial and lateral tibial slopes varied across the tibial slope, both among subjects and between the left and right sides of the same patient. The results presented herein supported these hypotheses, showing a variation in the tibial slope across the tibial plateau as a function of the distance from a sagittal or frontal reference plane. The proposed Matlab-based image processing method was effective at efficiently measuring MTS, LTS, and CTS across all slices (around 120 slices in each anatomical plane) with a time to completion of less than 20 s for a complete image stack. 

Anterior cruciate ligament (ACL) rupture frequently results from non-contact mechanisms such as anterior shear forces coupled with knee abduction moment during dynamic activity [26,27]. As previously established, non-contact ACL injuries are more prevalent in females than in their matched male counterparts [26]. Additionally, osteoarthritis (OA) of the knee has been shown to follow acute traumatic knee injury, but is also commonly associated with the aging process. Differences in the tibial slope between males and females have been identified in the literature and may be implicated in tibiofemoral joint arthrokinematics [10,12]. The geometric complexity of the proximal tibia and its implication in functional biomechanics of the knee joint both in weight-bearing and functional movement may contribute to increased risk of non-contact ACL injury and OA. 

Aside from implications in risk stratification and mitigation of non-contact ACL injuries and the development and progression of knee OA, PTS has implications in improving the effectiveness of total knee arthroplasty (TKA), therein restoring more optimal kinematics and quality of life post-operatively. In a secondary analysis of patients from two previous randomized controlled trials, Bellemans et al. found a significant correlation between range of motion following TKA and PTS [28]. Similarly, Malviya et al. reported similar findings in their secondary analysis of two controlled randomized studies that evaluated the range of motion for two similar total knee replacement systems [29]. Note, however, that Bellemans et al. reported an average increase of 1.7° flexion for each degree of increased posterior tibial slope, while Malviya et al. reported a 2.6° increase. Kansara and colleagues, on the other hand, were not able to demonstrate increases in flexion or functional outcomes regardless of the change made to the PTS [30]. In their study, Kansara et al. employed a method to measure tibial slope after TKA implantation similar to the methodologies explained by Hashemi et al. [10,12]. These variations in the impact of PTS on the restoration of effective range of motion following TKA may be due to inconsistencies in the measurement of tibial slope or differences in the surgical techniques required to implant specific arthroplasty systems.

Several authors [1,9,10,12,14,31,32] have reported on the tibial slope measured for patients via methods similar to those described by Hashemi et al. [12], excluding Hudek et al. [18] who used a method they described which relied on fitting two best-fit circles to the cortex of the proximal tibia and generating a reference axis from the midpoints of these circles. The range of the mean medial tibial slopes in the ACL-injured populations of these studies was 4.7°–11.2°, while the range for the uninjured controls was 4.1°–9.9° [1,9,10,12,18,31,32]. Only Stijak et al., Hashemi et al., and Hudek et al. reported on the lateral tibial slope with a range for the injured knees of 5.6°–9°, while the uninjured subjects ranged from 4.4°–6.0°. It cannot be said with certainty that these ranges are entirely related to the choice of measurement method for the tibial slope; however, as Faschingbauer et al. demonstrated in their study [19], there is a limited effectiveness in determining the tibial slope from a single 2D planar method. These studies also failed to address any variations in the tibial slope across the plateau, which, as we have shown in the present study, can vary greatly between and within the same subject. 

An obvious limitation to this study is that patient orientation in the scan FOV should be fairly consistent to the standard supine lying position used herein. To assess the effects of image orientation in the scan FOV the patients would have to be scanned in different positions and then analyzed with the methodology outlined above. However, the effects of slight variations of position within the scope of the supine lying position should be minimal on the resultant slopes calculated. Additionally, utilization of the “best-fit” feature within Matlab to reposition any misaligned tibia could be used to orient each in a more uniform fashion. This would ensure that all measurements were taken from the same reference position.

Future efforts based on the present work include expanding the limited sample population (*n* = 9) to include a larger cohort of subjects that can be used to identify differences in tibial slopes among men and women, ethnicities, age groups, and anthropometric percentiles. Simulation via finite element methods of the effect of critical tibial slope values on ACL strain, cartilage contact pressures, and stresses on other structures of the knee joint can help further the understanding of anatomic variation on ACL injury risk and the development and progression of osteoarthritis. To the authors’ knowledge, a tibial slope has not yet been identified that is indicative of these pathologies and injury mechanisms outside of “increased” slope leading to injury or disease, and a more concrete evaluation of the anatomy may prove useful in determining the root cause and optimal treatments therein.

Our current work has shown that the tibial slope is not a uniform structure, with variation across the tibial plateau, and assumptions with respect to constraining the cutting plane to any one given point may produce variation in the reconstructed tibial slope. Although a paradigm shift in the measurement of PTS clinically may be a monumental undertaking, further exploration and evaluation of alternative methods to the 2D planar measurement of the tibial slope is warranted with a larger sample size. Differences in the tibial slope between the sexes, population percentiles, age groups (infant, adolescent, adult), and activity levels (athletes, middle-aged adults, and older adults) could be elucidated that allow for development and innovation in surgical intervention, physical therapy, and preventative measures to reduce the risk of ACL injury, the development and progression of knee OA, and the effectiveness of TKA.

## Figures and Tables

**Table 1:** The mean (standard deviation) lateral, medial, and coronal tibial slopes, averaged and in the identified concavity zones for each subject.

Subject	Sex	Avg. LTS	Lat. Conc.	Avg. MTS	Med. Conc.	Coronal Avg.
Subject 1	F	14.8° (3.5)	18.5° (2.2)	13.2° (1.5)	13.5° (3.2)	0.4 (0.2)
Subject 2	F	5.4° (1.1)	5.5° (0.2)	5.7° (2.1)	4.4° (3.9)	0.4 (0.2)
Subject 3	F	3.2° (1.6)	4.4° (1.3)	7.6° (1.4)	8.1° (1.8)	0.4 (0.2)
Subject 4	F	8.7° (3.1)	9.5° (1.8)	10.1° (1.6)	11.0° (3.6)	0.4 (0.2)
Subject 5	F	8.1° (4.8)	7.2° (1.5)	15.6° (0.6)	15.7° (1.7)	0.4 (0.2)
Subject 6	F	3.4° (0.3)	3.3° (0.3)	5.9° (0.8)	5.3° (0.5)	2.4 (1.6)
Subject 7	M	−0.2° (0.9)	−0.2° (1.2)	2.3° (0.7)	2.8° (1.1)	2.4 (2.8)
Subject 8	M	3.3° (2.2)	3.9° (1.0)	3.3° (1.1)	3.6° (1.2)	0.4 (0.2)
Subject 9	M	2.2° (1.5)	3.1° (1.0)	5.1° (1.5)	5.2° (1.6)	0.4 (0.2)
